# Emerging Roles of Gut Virome in Pediatric Diseases

**DOI:** 10.3390/ijms22084127

**Published:** 2021-04-16

**Authors:** Valerio Fulci, Laura Stronati, Salvatore Cucchiara, Ilaria Laudadio, Claudia Carissimi

**Affiliations:** 1 Department of Molecular Medicine, Sapienza University of Rome, 00161 Rome, Italy; valerio.fulci@uniroma1.it (V.F.); laura.stronati@uniroma1.it (L.S.); 2 Department of Women’s and Children’s Health, Sapienza University of Rome, 00161 Rome, Italy; salvatore.cucchiara@uniroma1.it

**Keywords:** virome, metagenomics, children, inflammatory bowel disease, type 1 diabetes, malnutrition, diarrhea, celiac disease

## Abstract

In the last decade, the widespread application of shotgun metagenomics provided extensive characterization of the bacterial “dark matter” of the gut microbiome, propelling the development of dedicated, standardized bioinformatic pipelines and the systematic collection of metagenomic data into comprehensive databases. The advent of next-generation sequencing also unravels a previously underestimated viral population (virome) present in the human gut. Despite extensive efforts to characterize the human gut virome, to date, little is known about the childhood gut virome. However, alterations of the gut virome in children have been linked to pathological conditions such as inflammatory bowel disease, type 1 diabetes, malnutrition, diarrhea and celiac disease.

## 1. Introduction

Mammals are colonized by numerous microbes, collectively referred to as the microbiota. The microbiota forms a stable symbiotic relationship with the host and is indispensable for health maintenance. The most extensively investigated microbial community hosted by the human body is certainly the gut microbiome. Indeed, the gastrointestinal tract is the most densely populated microbial niche of the human body. Gut microbiota functions as a virtual organ participating in multiple physiological processes, such as immune development, nutrition and metabolism [1]. Perturbations of the complex structure of commensal communities, referred to as dysbiosis, can lead to the development of various pathological conditions, such as chronic noncommunicable diseases including atopies, metabolic syndrome, inflammatory bowel disease, cancer and some behavior disorders [2,3,4,5,6,7].

Although the eubacteria are the best characterized component of the human gut microbiota, it also includes commensal populations of viruses, fungi, multicellular parasites and archaea [8,9]. In this review, we will use the term “microbiome” referring to the bacterial communities, not including in this definition viruses and unicellular eukaryotes or archaea.

Over the last decades, microbiota studies have focused primarily on the characterization of the microbiome, mainly due to methodological limitations. Indeed, several biochemical and bioinformatic methods have been largely developed to study the microbiome structure and function [10,11], compared to methodologies aimed at the study of the viral community, hereinafter referred to as virome. Nevertheless, evidence highlights that the virome, consisting in DNA and RNA eukaryotic viruses, bacterial viruses (i.e., bacteriophages) and archaeal viruses, exerts a fundamental role in the host wellbeing [12,13].

Although shotgun metagenomics has been successfully applied to investigate the diversity of microbial and viral communities living in different environments, spanning from marine ecosystems [14,15] to the human body [16,17,18], the human virome remains largely uncharacterized. Nevertheless, recent studies underline the ability of the virome to dynamically interact and shape the microbiome [19,20].

During infancy and childhood, a dynamic reciprocal equilibrium between bacterial and viral communities of the gut is established [21]. Although further confirmation is needed, available data suggest that these communities remain mostly stable throughout the entire adulthood of each individual [22]. Interestingly, the adult virome alterations have been linked to increased disease susceptibility [23,24,25], suggesting that maintaining or restoring a structurally and functionally correct composition of the virome might represent in the near future a promising approach to prevent or cure different human diseases [26,27].

**Figure 1:**
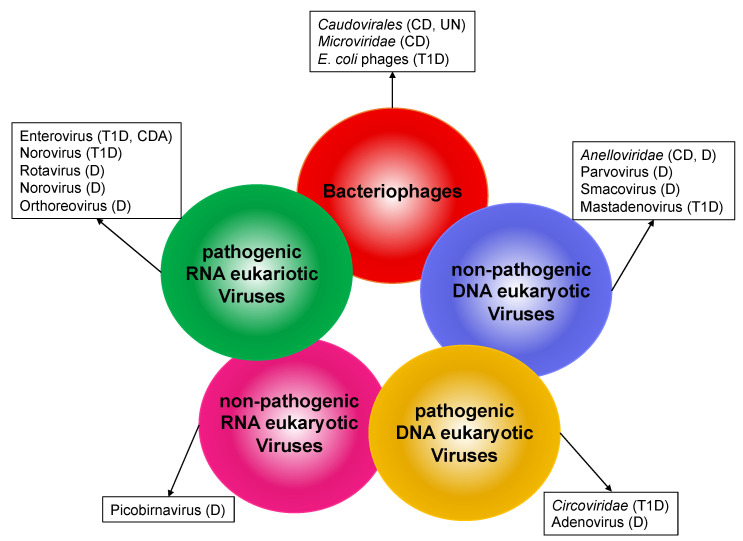
Alteration of human gut virome in pediatric diseases. Circles represent components of the human gut virome. Viruses or virus families associated with different pathological conditions in children are listed in the boxes. CD (Crohn’s disease); UN (undernutrition); T1D (type 1 diabetes); D (diarrhea); CDA (celiac disease autoimmunity).

Although the knowledge of childhood virome is currently very limited, modifications of the virome composition have been also associated with several diseases during the pediatric age [28] (Figure 1). Thus, research into virome differences in pediatric patients may help progress our understanding of the etiology of childhood diseases. Here, we will review the current knowledge about the human gut virome in childhood health and disease.

## 2. Composition of the Human Gut Virome

The human gut virome consists of three main components:

- Bacteriophages
- DNA eukaryotic viruses
- RNA eukaryotic viruses

### 2.1. Bacteriophages

Bacteriophages are a wide clade of viruses infecting bacterial cells. Bacteriophages may exploit two main infection strategies: in the lysogenic cycle, phages integrate their genome into the bacterial host chromosome and are replicated synchronously with the bacterial genome; in the lytic cycle, instead, the viruses hijack the transcription/translation apparatus of the host prokaryotic cell to produce viral particles which are ultimately released either chronically or upon host cell lysis. Bacteriophages are an extremely diverse clade; each virus can exploit different combinations or flavors of the two main strategies with complex patterns depending also on environmental conditions [29]. The most abundant groups of bacteriophages in the adult human gut belong to the order of *Caudovirales* (including *Myoviridae*, *Podoviridae*, *Siphoviridae* and the recently discovered clade of crAss-like phages) and the family of *Microviridae* [22]. Importantly, different subclades of bacteriophages have differential tropism towards prokaryotic species.

### 2.2. DNA Eukaryotic Viruses

The human gut virome includes both single-stranded DNA viruses (*Anelloviridae*, *Circoviridae*) and double-stranded DNA viruses (*Adenoviridae*, *Herpesviridae*, *Papillomaviridae*, *Polyomaviridae*) [20]. Some of these clades are not associated with clinically relevant infections (i.e., *Anelloviridae*), despite being able to proficiently infect human cells. On the contrary, members belonging to each of the double-stranded DNA virus families are associated with clinically relevant infectious diseases.

### 2.3. RNA Eukaryotic Viruses

Finally, several human gut viruses are RNA viruses able to infect eukaryotic cells, including pathogenic and nonpathogenic viruses. The most abundant families are the nonpathogenic *Picobirnaviridae* and *Virgaviridae* (a clade of plant viruses), suggesting that a relevant fraction of intestinal RNA virus primarily derives from the diet [17]. On the other hand, the less copious RNA virus families of *Reoviridae* (including the Rotavirus genus), *Caliciviridae* (including the Norovirus genus) and *Picornaviridae* (including the Enterovirus genus) are commonly associated with gastroenteritis [30,31,32].

## 3. Diversity and Dynamics of the Gut Virome in Children

The composition of the childhood virome was first investigated in the seminal work by Breitbard and colleagues [33], who exploited shotgun metagenomics to assess the gut virome composition of a one-week-old infant. Although a large proportion (66%) of the reads sequenced in this report did not match any annotated sequence, about half of the hits mapping to known Genbank references belong to the group of bacteriophages. The authors compared the abundance of the identified viral genomes in the gut virome of one infant at the age of one- and two-weeks, highlighting a very fast change of the infant gut virome composition. Interestingly, this infant gut virome showed a much lower diversity as compared to adult samples. Further investigation confirmed these findings, highlighting rapid virome composition changes in the first two years of life. Interestingly, a recent paper which examined longitudinal changes in the virome of infants within the first year of life showed that viral richness is lower in the earliest-in-life specimens compared to the latest timepoint [34].

By the age of two, the eukaryotic virus family of *Anelloviridae* becomes the major component of the gut virome [21]. However, this latter finding is at odds with several reports suggesting that bacteriophages account for the vast majority of viruses in the adult gut virome [35,36,37]. These data may be reconciled by the observation that a transient increase in eukaryotic viruses during childhood could be ascribed to an immature immune system [38]. Once a fully competent immune response has been achieved, the load of eukaryotic viruses is accordingly largely tamed by the adaptive immune response.

The increase in eukaryotic viruses observed in the first two years is mirrored by a reduction of the bacteriophage richness and diversity. Interestingly, in the gut, the bacteriophage decrease is paralleled by a significant expansion of bacterial diversity and richness, suggestive of a prey-predator dynamic [21]. In a longitudinal study in a cohort of 22 individuals, a set of age specific contigs was identified, suggesting that virome development during infancy proceeds through different steps characterized by specific viromes [39].

It is largely accepted that the microbiome of the infant gut is largely determined by vertical transfer from the mother, with a major contribution from maternal gut microbiome [40]. Nevertheless, Maqsood and colleagues reported a rather poor correlation between mother and infant gut virome, as compared to the one observed for the microbiome [41]. However, since this study is focused on the analysis of fecal samples collected in the first four days after birth, it cannot be ruled out that the gut virome of an older infant might display a greater correlation with the one of the mothers. Indeed, one might reasonably expect that the microbiome, which is to a large extent shared with the mother, may shape by selection the virome. The observation that the newborn gut virome poorly overlaps the one by the mother pinpoints that other virus sources should be involved. A recent report suggests that the main reservoir of bacteriophages in the infant gut is represented by prophages integrated in the genomes of colonizing bacteria [42].

Maternal milk has been shown to contribute to the establishment of both infant gut microbiome and virome. Furthermore, the bacterial and viral content of mother’s milk significantly correlates with that of the infant gut [43]. Specifically, *Bifidobacterium*-infecting phages (Bifidophages) are transmitted vertically, most likely through maternal milk [44]. However, the exact origin of maternal milk microbiome and virome is still a matter of debate [45]. Breastfeeding also affects the colonization of the infant gut by eukaryotic viruses. Indeed, newborns exclusively fed on formula milk house more eukaryotic viruses in the gut compared with those fed partially or fully on breast milk [42].

Similarly, the mode of delivery impacts on the gut virome composition. Indeed, cesarean section delivered newborns show a lower abundance of *Anelloviridae* and temperate Biphidophages and different trans kingdom interactions between bacteria and bacteriophages as compared to vaginally delivered newborns [41,46].

## 4. Alterations of Gut Virome in Childhood Diseases

### 4.1. Inflammatory Bowel Disease

Microbiome changes have been associated with inflammatory bowel disease (IBD), complex disorders including Crohn’s disease (CD) and ulcerative colitis (UC), and represent a potential diagnostic or prognostic tool in this context. Hence, the use of probiotics has been explored as a potential therapeutic intervention in IBD [25,47,48,49].

Although the incidence of pediatric IBD is steadily increasing [50], a limited number of studies addressed the potential role of the gut virome in early and very early onset IBD. Recently, several groups carried out multi-omics research to comprehensively assess the composition of both the microbiome and the virome in the gut of pediatric [51,52] and adult IBD patients [25]. Surprisingly, the microbial dysbiosis that is a well-known correlate of IBD does not seem to be paralleled by major changes in the gut virome. Accordingly, fecal microbiota transplantation (FMT) had a higher impact on the gut microbiome than on the virome in UC patients [51].

Fernandes and colleagues examined the fecal virome of children with CD, UC and healthy controls. They reported an association of *Caudovirales* with CD rather than UC and a reduced richness of *Microviridae* strains in CD relative to healthy controls in pediatric patients [53]. Accordingly, a later study focused on very early onset IBD reported a higher ratio of *Caudovirales* to *Microviridae* in these patients as compared to controls, along with an increase in *Anelloviridae*, a family of eukaryotic viruses infecting mammalian cells [54]. These findings are in agreement with those previously reported in adult IBD [55]. Since in both cases the increase of the *Anelloviridae* is positively associated with the immunosuppressive therapies but not with disease activity, one might infer that this phenomenon is a side effect due to immunosuppression [54,55]. Further investigation will be required to assess whether the higher *Caudovirales* to *Microviridae* ratio has a role in reshaping the bacterial populations or is rather a secondary effect due to the dysbiosis in IBD.

### 4.2. Type 1 Diabetes

Type 1 diabetes (T1D) is a chronic disease due to the autoimmune mediated destruction of pancreatic islet β cells. The clinical onset of T1D is often preceded by islet autoimmunity. This preclinical phase is defined by the presence of β cell autoantibodies directed against insulin, glutamic acid decarboxylase 65, insulinoma antigen-2 or ZnT8 transporter [56]. Genetically susceptible individuals develop the disease presumably in response to a combination of environmental triggers which have not been conclusively identified thus far [57]. Viral infections have been repeatedly proposed as one of the possible causes of the disease development [58,59,60]. A meta-analysis based on a large number of studies has reported a significant association between Enterovirus infection and T1D onset [61]. Mechanistically, it has been suggested that viral replication in the pancreas might result in chronic infection of islet β cells [62]. This could be due to a less efficient antiviral response of β cells to infection with diabetogenic viruses than do α cells, thus explaining why pancreatic β cells, but not α cells, are targeted by an autoimmune response and killed during the development of T1D [63].

Nevertheless, the studies aimed to characterize the gut virome in T1D patients conducted to date have yielded partially contradictory results. A pioneering work on a cohort of 38 children (19 who developed islet autoimmunity and 19 matched controls) did not identify any clear association between the gut virome composition and the onset of islet autoimmunity [64]. A later longitudinal NGS virome study on 22 children (11 T1D patients vs. 11 matched controls) observed an association between *Circoviridae*-related sequences and control samples, but no correlation between enterovirus and T1D. Furthermore, the authors reported an increased Shannon diversity in the bacteriophage population in controls [39].

Otherwise, a NGS gut virome analysis on a cohort of 45 patients diagnosed with islet autoimmunity and 48 age-matched controls reported a significant association of enterovirus with islet autoimmunity. Notably, the authors also reported that norovirus was only detected in control subjects [65]. Shortly after, convincing evidence that norovirus infection has a significantly protective role against the development of T1D has been reported [66].

Up to date, the largest study on infant gut virome was performed by the TEDDY (The Environmental Determinants of Diabetes in the Young) Study Group [67]. Authors prospectively analyzed the virome of 383 children with islet autoimmunity and 112 children with T1D (along with nested-matched paired controls), by collecting samples monthly, from the age of 3 months until the detection of either condition. They found that prolonged Enterovirus B infection preceded subsequent initiation of islet autoimmunity in children, but not T1D. The viral subgroup found to be significantly linked to islet autoimmunity was Coxsackievirus B (CVB), suggesting that it could have properties that make it potentially diabetogenic. On the other hand, human Mastadenovirus C (HAdV-C) was detected in fewer children who developed either islet autoimmunity or T1D than matched controls. Intriguingly, the authors suggest that early HAdV-C infections before the age of 6 months were associated with a low risk of islet autoimmunity because HAdV-C and CVB compete for the same receptor, i.e., cell-surface coxsackie and adenovirus receptor (CXADR), which is highly expressed in β cells.

Finally, a recent re-analysis of publicly available data from a previous longitudinal microbiome study stressed that a re-activation of temperate *Escherichia coli* bacteriophages, paralleling a reduction in the abundance of *E. coli*, could contribute to the T1D development. The authors speculate that this phenomenon could lead to bacterial amyloid release, which in turn could trigger islet autoimmunity [68].

The contradictory evidence resulting from various gut virome studies can be partially explained by the dramatic improvement of the NGS virome sequencing technology and analysis tools over the last decade. Indeed, in recent studies, a significant improvement in comprehensiveness and sensitivity of eukaryotic virus detection has been attained through the development of target-enrichment protocols. In these approaches, viral sequences are enriched in sequencing libraries through viral genome hybridization capture, such as VirCapSeq-VERT [69] or ViroCap [70]. Moreover, the lack of extensive viral sequences databases may have hampered the analysis of the earlier dataset, which therefore may have failed to capture differences highlighted instead by later reports.

Furthermore, different viral-like particles (VLP) isolation protocols and/or library preparation protocols may have led to a differential recovery of eukaryotic rather than prokaryotic viruses or RNA rather than DNA viruses [36].

### 4.3. Undernutrition

Undernutrition is a global health challenge, involving up to 25% of children worldwide under the age of five. Evidence suggests that malnutrition is likely associated with alteration in the intestinal microbiome [71,72]. Furthermore, FMT from undernourished children into murine models is causative of growth defects, which can be attenuated by FMT from healthy children [73]. Interestingly, *Lactobacillus plantarum* supported juvenile growth in germ-free mice. Mechanistically, *Lactobacillus plantarum* sustains growth hormone activity thus overcoming growth hormone resistance, a consequence of chronic undernutrition [74].

The virome of undernourished children has been investigated in a longitudinal study on Malawian twin pairs, highlighting specific changes of the virome composition in children affected by Severe Acute Malnutrition (SAM). Data highlighted a set of viral contigs significantly associated with the disease. The authors confirm that virome composition is quite stable within each individual. Notably, discordant twin pairs (i.e., only one of the two twins displayed a malnutrition phenotype) shared a malnutrition-associated virome signature, suggesting that virome alterations may precede the development of SAM [75]. Indeed, the growth velocity of a different cohort of Malawian children is associated with a set of bacteriophages, but not with eukaryotic viruses [76]. Accordingly, specific association between a gut bacteriophages community and stunted children was reported. Interestingly, in vitro experiments demonstrated that these phages are able to reshape the bacterial community of non-stunted children, suggesting that they can actually affect the composition of the gut microbiome of their hosts, potentially contributing to their phenotype [77].

### 4.4. Diarrheal Diseases

Globally, diarrheal diseases are the fifth leading cause of death in children younger than five years, causing nearly half a million deaths [78], the majority of which occur in non-industrialized countries, such as Southeast Asia and sub-Saharan Africa [79]. While mortality and morbidity have been significantly reduced in the last decade, diarrhea caused by viral and bacterial infections is still a major public health problem in developing countries [78].

Enteric viruses, namely eukaryotic viruses infecting the gut play a major role in causing diarrhea in children. This group comprises RNA viruses such as Rotaviruses, Noroviruses, Astroviruses, Reoviruses, Enteroviruses and retroviruses, as well as DNA viruses such as Adenoviruses. All of them are considered part of the human gut virome, and although rare, they can be detected also in the gut of healthy infants [39,80,81].

Rotavirus is the leading etiological agent of diarrhea mortality in children under the age of five (27% of diarrhea cases), followed by Adenovirus (11%) and Norovirus (2%) [78]. In addition, other viruses are known to cause diarrheal diseases, such as human Astrovirus, Sapovirus and several viruses belonging to the *Picornaviridae* family [82]. 

It is worth mentioning that many diarrhea episodes still remain unexplained, as no etiological agent is still determined. Some of them might be due to known viruses, previously unlinked to diarrhea, for which no tests were performed, and some to still unknown viral agents. In the last decade, the use of metagenomics based on NGS techniques, allowing simultaneous detection and genomic characterization of the entire viral population in a sample, enabled researchers to identify both known and previously unknown viruses as new etiological agents in diarrhea in children.

Thereby, a new family of Parvovirus, which was named Bufavirus, was identified in fecal samples from children affected by diarrhea of unknown etiology in Burkina Faso [83]. Even though their role in the pathogenesis of acute diarrhea still remains unclear, Bufavirus genomes have been further detected and characterized in the stool of children with diarrhea in Bhutan, Turkey, Thailand and Tunisia [84,85,86,87]. Furthermore, in a study including children with unexplained gastroenteritis from the The Netherlands, a new Picobirnavirus was identified and placed in the new genogroup III [88]. Interestingly, a new clade of small circular single-stranded DNA viral genomes was genetically characterized in fecal samples collected from Peruvian children with diarrhea of unknown origin, namely “Pecoviruses” (Peruvian stool-associated circo-like viruses) [89].

Metagenomics has also proved very useful in identifying known viruses previously not associated with pediatric gastroenteritis and diarrhea. Indeed, gut virome analysis in Cameroonian children with unexplained diarrhea reveals the presence of known viruses previously never associated with diarrheal diseases such as Picobirnavirus, Anellovirus and Smacovirus [85]. As well, viruses that are uncommon causes of gastroenteritis in humans, such as the mammalian Orthoreovirus, were identified in the gut virome of children with diarrhea in Cameroon [90] and Brazil [91].

### 4.5. Celiac Disease

Celiac disease is an autoimmune enteropathy triggered by the ingestion of gluten, commonly beginning in early childhood. A subclinical or preclinical phase, the so-called celiac disease autoimmunity (CDA) is characterized by the appearance of autoantibody against tissue transglutaminase, which deamidates gluten-derived gliadin peptides [92].

Celiac disease has a strong genetic predisposition. However not all the genetically predisposed children developed the disease, suggesting that additional environmental triggers are involved in the pathogenesis of celiac disease. Several studies have suggested a possible role of viral infections in the disease pathogenesis. In particular gastrointestinal infections of Adenovirus, Enterovirus, Rotavirus and Reovirus in early life have been associated with increased risk of later celiac disease [93,94,95,96,97]. Recently, a prospective metagenomic screening of fecal virome investigated the effects of viral and gluten exposures prior to development of CDA in genetically predisposed children (88 CDA children and matched controls) [98]. Frequent exposure to Enterovirus between one and two years of age was associated with higher risk of CDA. Moreover, the risk of CDA was increased in the highest among Enterovirus positive children who had the highest gluten intake, indicating a cumulative effect of these two factors in the development of CDA in genetically at-risk children. Overall, although these studies do not evaluate pathogenetic mechanisms of early-life infections and CDA development, they offer new insights for the improvement of prevention in celiac disease.

## 5. Future Directions

Despite extensive efforts in the characterization of the human virome, current knowledge of this issue is still limited. The lack of a comprehensive view of the gut virome in childhood is, at least partially, due to the small number of reports based on large cohorts of samples. However, methodological and computational challenges are to be addressed, yet.

Pipelines for virome shotgun data analysis are still being developed. In metagenomics, the assembly of reads into genomes to yield a reliable genomic database for subsequent mapping of reads to taxonomic groups plays a key role. While dramatic progress has been achieved in the last decade in the bacterial genome assembly, in particular for the heavily investigated niches, such as the human gut microbiome, the development of dedicated software to proficiently assemble viral reads is still an ongoing process [99]. The intrinsic features of viruses (lack of a consensus definition of “species”, large amount of reads still mapping to “viral dark matter”, complex and in some cases still poorly understood life cycles, retrieval of reads cross-mapping to several viral genomes, presence of viral relics in eukaryotic genomes) largely contribute to these challenges.

The first studies were affected by a lack of extensive databases allowing mapping of reads to the corresponding viral genomes, and this resulted in an overall mapping of viral reads smaller than 30%. In these cases, most of the information that was collected could not be exploited.

The shortcomings of earlier and naive approaches exploiting simple matches to databases as a mean for taxonomic assignment have been highlighted. Those approaches may in fact lead to inconclusive or biased outcomes [100]. Furthermore, the use of innovative methods for the classification of viral sequences has proven to be effective in the reanalysis of previously collected datasets, likely yielding more robust data [101].

Since in the last decade several reports suggesting a role of the infant gut in different clinical disorders have been collected, the use of more recent algorithms for the reanalysis of published datasets could lead to further potentially significant observations which escaped earlier investigations.

## Data Availability

Not applicable.
